# Cardiac patients’ perceptions of neighboring patients’ risk: influence on psychological stress in the ED and subsequent posttraumatic stress

**DOI:** 10.1186/s12873-017-0144-3

**Published:** 2017-11-06

**Authors:** Beatrice Konrad, David Hiti, Bernard P. Chang, Jessica Retuerto, Jacob Julian, Donald Edmondson

**Affiliations:** 1 Center for Behavioral Cardiovascular Health, Columbia University Medical Center, 622 W. 168th St, PH9-317, New York, NY 10032 USA; 2 Division of Emergency Medicine, Columbia University Medical Center, New York Presbyterian Hospital, 622 West 168th Street, New York, NY 10032 USA

**Keywords:** Emergency department, Posttraumatic stress disorder, Threat perception, Acute care environment, Crowding, Acute coronary syndrome

## Abstract

**Background:**

As many as 12% of acute coronary syndrome (ACS) patients screen positive for post-traumatic stress disorder (PTSD) symptoms due to their cardiac event, and emergency department (ED) factors such as overcrowding have been associated with risk for PTSD. We tested the association of patients’ perceptions of their proximity to a critically ill patient during ED evaluation for ACS with development of posttraumatic stress symptoms (PSS) in the month after hospital discharge.

**Methods:**

Participants were enrolled in the REactions to Acute Care and Hospitalization (REACH) study during evaluation for ACS in an urban ED. Participants reported whether they perceived a patient near them was close to death. They also reported their current fear, concern they may die, perceived control, and feelings of vulnerability on an Emergency Room Perceptions questionnaire. One month later, participants reported on PTSD symptoms specific to the cardiac event and ED hospitalization.

**Results:**

Of 763 participants, 12% reported perceiving a nearby patient was likely to die. In a multivariate linear regression model [F(9757) = 19.69, *p* < .001, R^2^ adjusted = .18] with adjustment for age, sex, GRACE cardiac risk score, discharge ACS diagnosis, Charlson comorbidity index, objective ED crowding, and depression symptoms at baseline, perception of a nearby patients’ likely death was associated with a 2.33 point (95% CI, 0.60–4.61) increase in 1 month PTSD score. A post hoc mediation analysis with personal threat perceptions [F(10,756) = 25.28, *p* < .001, R^2^ adjusted = .24] showed increased personal threat perceptions during the ED visit, B = 0.71 points on the PCL per point on the personal threat perception questionnaire, β = 0.27, *p* = .001, fully mediated association of participants’ perceptions of nearby patients’ likely death with 1-month PTSD score (after adjustment for ED threat perceptions,) B = 0.89 (95% CI, −1.33 to 3.12), β = 0.03, *p* = .43, accounting for 62% of the adjusted effect and causing the main effect to become statistically nonsignificant.

**Conclusions:**

We found patients who perceived a nearby patient was likely to die had significantly greater PTSD symptoms at 1 month. Awareness of this association may be helpful for designing ED patient management procedures to identify and treat patients with an eye to post-ACS psychological care.

## Background

Emergency departments (ED) can be extremely stressful environments for patients, particularly when a patient’s condition is potentially life-threatening. Patients being treated for an acute coronary syndrome [ACS; non-ST elevation myocardial infarction (NSTEMI) and unstable angina (UA)] often report feelings of fear, vulnerability, loss of control, and worry about their risk of imminent death [1]. Fear of death during a potentially traumatic event is one of the strongest predictors of subsequent posttraumatic stress disorder (PTSD), and witnessing the death of others can induce PTSD even in individuals who are not themselves at risk [2, 3].

A recent meta-analysis found that as many as 12% of ACS patients screen positive for PTSD symptoms due to their cardiac event [1], and PTSD following ACS evaluation is associated with increased risk of recurrent cardiac events, poor quality of life, poor medication adherence, and mortality [4–7]. A number of environmental factors in the ED may influence patient’s perception of life threat, and increase risk for subsequent PTSsD in ACS patients. For example, ED overcrowding [1] and the perception of poor doctor-patient communication in the ED have been associated with PTSD symptoms after ACS evaluation [8].

While in the ED, patients may also witness death or serious illness in others around them, which may increase PTSD risk. Studies of natural disasters including massive earthquakes [9–11] have found that witnessing death increases PTSD risk substantially. Similarly, survivors of Hurricane Katrina who saw corpses during the disaster were at nearly twice the risk for clinically significant PTSD symptoms (OR = 1.72) relative to survivors who had not seen them [3]. This association has been documented in healthy individuals who have witnessed death in more normative settings as well. For example, one study found high PTSD symptoms in family members who witnessed an unsuccessful out-of-hospital cardiopulmonary resuscitation [12].

The Enduring Somatic Threat (EST) model provides a theoretical basis for understanding PTSD due to acute life-threatening cardiovascular events, and its third proposition is that the underlying source of distress that causes PTSD is the terrifying awareness of mortality that is made conscious by the event. The threat of mortality may become normalized for emergency medicine clinicians, but for many patients in the ED, perceiving that other patients in the ED might be close to death may be highly stressful, and may increase their own feelings of vulnerability. Indeed, a recent study showed a significant impact of in-hospital critical illness events on risk for subsequent critical illness events in nearby patients, a finding that demonstrated that the experience and outcomes of patients near one another are interconnected [12, 13]. However, no study to date has tested the influence of patients’ perceptions of nearby patient acuity in the ED on subsequent psychological outcomes.

The present study is the first to test the association of patient perceptions of mortality risk in nearby patients with subsequent PTSD symptoms after ED evaluation for a potentially life-threatening ACS. We hypothesized that participants who perceived that neighboring patients were likely to die would report greater PTSD symptoms due to the ACS event 1 month after discharge, and that the effect would be at least partially mediated by participants’ greater perceptions of their own personal vulnerability during ED evaluation.

## Methods

Data collection procedures and measures used in the Reactions to Acute Care and Hospitalization (REACH) study are summarized in brief below. Detailed definitions and explanations of measures and covariates have been published previously [14].

### Procedures

Participants were enrolled in the REactions to Acute Care and Hospitalization (REACH) study during evaluation for ACS in an urban ED (Columbia University Medical Center in New York City). Patients were potentially eligible for participation once given a diagnosis of “probable ACS” by treating ED physicians. In the ED, participants reported on whether they perceived that a patient near them was likely to die. They also reported on their current fear, concern that they may die themselves, perceived control, and feelings of vulnerability on a 6-item Emergency Room Perceptions questionnaire. During a second interview, either during inpatient stay or by telephone (if discharged prior to follow-up) a median of 3 days later, participants reported on pre-hospital depression symptoms and were asked to recall their ED experience using identical questionnaires framed in the past tense. One month after enrollment, participants reported on PTSD symptoms specific to the cardiac event and ED evaluation/hospitalization by phone.

### Measures

#### PTSD symptom score

The PTSD Checklist-Specific for ACS [PCL-S] [15] is a 17-item checklist based on DSM-IV criteria for PTSD symptoms [16]. The PCL-S queried PTSD symptoms in response to the “heart problem, ED visit, and hospitalization” that occurred when the subject enrolled in the study, on a 5-point Likert scale (0, not at all, to 4, extremely). The PCL-S has been shown to have good internal consistency (Cronbach’s α = 0.94 [15]), and test-retest reliability at 2–3 days and 1 week [15]. Because the DSM 5 and corresponding PCL 5 were released during the study period, participants who were enrolled after release of the PCL 5 completed that questionnaire with respect to the “heart problem, ED visit, and hospitalization.” For the present analyses, we used only the PCL 5 items (items 1–9, 12–15, 18–20) that correspond to PCL-S (DSM-IV) items, and adjusted scores such that they were on the same scale.

#### Nearby patients item

During the ED enrollment interview, participants reported on their perceptions of mortality risk in proximal patients: “Does it seem like another patient in the emergency room may die?” on a Likert scale (0, not at all, to 3, extremely). We dichotomized participant responses into 1 = score of 2 (moderately) or 3 (extremely) or 0 = score of 0 (not at all) or 1 (a little bit).

#### Threat perception during ED stay

During the same ED interview, patients reported their perceptions of personal threat on a 6-item questionnaire. Patients were asked to rate on a 5-point Likert scale ranging from 0 (not at all) to 4 (extremely) agreement with statements such as “I am afraid,” “I feel helpless,” “I feel vulnerable,” “I worry that I may die,” “I believe this will be a big event in my life,” and “I worry that I am not in control of my situation.”

#### ED recall at 3 days post-enrollment

After enrollment, participants recalled their perceptions of nearby patients and their own personal threat during their ED stay using identical items to those administered in during ED enrollment, but keyed to the ED stay during which they were enrolled.

### Covariates

Covariates used in analysis were discharge ACS status, GRACE cardiac risk score, Charlson comorbidity index, baseline depressive symptoms, and objective ED crowding at initial evaluation using emergency department work index (EDWIN) score.

### Statistical analysis

We hypothesized that participants’ perceptions of nearby patients’ likely death influenced their perception of their own personal threat. However, it is possible that participants’ perceptions of their own personal threat and other pre-existing vulnerabilities for PTSD may have influenced their perceptions of other patients. To minimize affective bias in participant reports, our grouping variable for exposure to high acuity patients was computed so that it reflected participants’ experience, was related to objective metrics that would be expected to correlate with the grouping variable, and was unrelated to pre-existing patient variables that might bias participant reports.

Because participants were enrolled during ED evaluation, most spent many hours in the ED after their study enrollment (and thus after responses to the items concerning nearby patients and concurrent personal threat were recorded). Therefore, we expected that during the 3-day follow-up interview, some patients may endorse recall for having been near a patient that was likely to die even though they had not endorsed it during the initial enrollment (i.e., it had not yet occurred when they were enrolled). However, if some patients endorsed the item in the ED, but not at the follow-up, we hypothesized that those endorsements were most likely to be influenced by participant factors, such as depression, that negatively influence participant perceptions. Therefore, we conducted a chi-square test of the bivariate association between endorsement of proximity to a dying patient assessed in the ED during enrollment with recall for that experience 3 days later (i.e., during your ED stay, did it seem as though a nearby patient might die?).

Once we chose the most valid assessment of participants’ perception of proximity to another patient who was likely to die, we tested the association of that variable with PTSD symptoms at 1 month. We tested a multivariate linear regression model with PTSD score as the dependent variable, and the dichotomous variable for perception that a nearby patient may die as the primary predictor variable. We adjusted for the demographic and clinical covariates listed above, and depression in the 2 weeks prior to enrollment. Our model was specified a priori, and conducted using direct entry of covariates in pre-specified blocks. Covariates were chosen based on prior research on contributors to PTSD risk in cardiac patients [17], as well as survivors of other types of potentially traumatic events [18]. Based on our power calculations for a model with 8 covariates, a sample size of 763 would allow us to detect a small effect size (<0.05) for our primary predictor, assuming power of 0.8 and an overall type I error rate of 0.05. In a post-hoc mediation analysis, we tested whether greater participant personal threat perceptions (i.e., perceptions of personal fear, lack of control, vulnerability, and mortality risk) accounted for the association of perceiving critical illness/mortality risk in nearby patients with subsequent PTSD symptoms. We tested the assumptions of linear regression, including normality of residuals, homoscedasticity, linearity of associations between predictors and the outcome variable, indicators of multicollinearity (variance inflation factor; VIF), and the influence of outliers. To ensure independence of observations, once a patient was enrolled, they could not be enrolled again even if they were re-hospitalized. None of the assumptions were significantly violated based on traditional guidelines, although PCL scores were positively skewed. Log transformation partially normalized the PCL distribution, but results did not differ substantially when we replaced the PCL with the transformed PCL score as the dependent variable, so results are reported in the original metric to aid interpretation.

## Results

Participants were 763 patients being evaluated for acute coronary syndrome in the ED. During the ED enrollment interview, 20% of subjects reported perceiving that a nearby ED patient was moderately or extremely likely to die. However, there was some disagreement between participants’ reports of being near a patient who was likely to die recorded in the ED versus at 3 day follow-up. We found that 12% of participants who recalled being near a patient that was likely to die did not endorse that item during ED enrollment (i.e., perhaps they had not yet been exposed at ED enrollment). More importantly, however, 44% of those who endorsed being near such patients during their ED stay did not endorse recall for being near a patient who was moderately or extremely likely to die during the follow-up interview. Therefore, we conservatively included participants in the “perceived a nearby patient as likely to die” only if they endorsed perceiving that a nearby patient was likely to die both during the ED enrollment interview and the 3 day follow-up recall. This change reduced the association of the nearby patient item with pre-existing depression from *r* = .13 to *r* = .09, and with personal threat perceptions in the ED from *r* = .26 to *r* = .20. Further, the more conservative variable was significantly associated with the mean EDWIN score (objective ED crowding) during participant’s ED stay, *r* = 0.10. All subsequent analyses used this more conservative criterion for grouping participants into those who had versus had not perceived a nearby patient who was likely to die.

Ninety-one participants (12%) met the more conservative criterion. Table 1 gives participant characteristics by this grouping variable. Participants who perceived a nearby patient as likely to die were younger, with higher depression, and were treated during periods that the ED was more crowded. Prior to adjustment for covariates, patients who perceived that a nearby ED patient was likely to die (endorsed at both time points) had 4.12 points [95% confidence interval (CI) = 1.67–6.47] greater PTSD score at 1 month post-discharge (*p* < .001). These data, along with subsequent covariate adjustment and post-hoc mediation test results, are listed in Table 2.

**Table 1:** Participant characteristics by report of nearby patient likely to die

	Perceived a nearby patient was likely to die	
	YES (*N* = 91)	NO (*N* = 676)
PTSD Score (1 month)**	28.3 (14.8)	24.2 (10.6)
Age*	58.0 (11.2)	61.7 (13.0)
Sex, Male (%)	52	54
Hispanic ethnicity (%)	58	56
Confirmed ACS** (%)	24	35
GRACE Risk Score	142.8 (49.7)	154.6 (47.2)
Charlson Comorbidity Index	1.7 (1.9)	1.9 (2.1)
PHQ depression score	8.1 (5.9)	6.3 (5.9)
Mean EDWIN crowding score**	1.58 (.41)	1.46 (.37)
Personal ED threat perceptions**	13.29 (5.25)	10.63 (4.09)

*SD* standard deviation

*p < .05; **p < .01

**Table 2:** Regression models predicting 1-month PTSD symptoms from proximity to dying patient, covariates, and participant threat

Variable	Bivariate association	*p*	Adjustment for covariates	*p*	Mediation test	*p*
Nearby patient likely to die	B = 4.117; β = .118	0.001	B = 2.334; β = .067	.044	B = .888; β = .026	.432
Age	–	–	B = −.082; β = −.094	.012	B = −.057; β = −.065	.074
Male	–	–	B = −.879; β = − .039	.248	B = −.908 β = −.040	.215
Hispanic	–	–	B = .537; β = .024	.472	B = .288; β = .013	.689
Charlson comorbidity score	–	–	B = .204; β = .038	.275	B = .263; β = .048	.144
GRACE cardiac risk score	–	–	B = −.004; β = −.019	.607	B = −.005; β = −.021	.554
Depressive symptoms (PHQ-8 score)	–	–	B = .752; β = .394	.000	B = .569; β = .298	.000
Confirmed ACS			B = .693 β = .029	.398	B = .459; β = .019	.561
EDWIN ED crowding score			B = 1.904; β = .063	.054	B = 1.559; β = .052	.102
Patient threat perceptions in the ED			–	–	B = .708; β = .272	.000

B = unstandardized regression coefficient; β = standardized regression coefficient

In the first multivariate linear regression model [F(9757) = 19.69, *p* < .001, R [2] adjusted = .18] with adjustment for age, sex, GRACE cardiac risk score, discharge ACS diagnosis, Charlson comorbidity index, EDWIN score, and continuous PHQ score at baseline, perception of a nearby patients’ likely death was associated with a 2.33 point (95% CI, 0.60–4.61) increase in PTSD score, β = 0.07, *p* = .04. The other significant predictors in that model were baseline depression score (B = 0.74 points per point on the PHQ, β = 0.39, *p* = .001), younger age (B = −0.08 points per year, β = −0.09, *p* = .01), and EDWIN score (B = 1.90 per point, β = 0.06, *p* = .05).

A post hoc mediation analysis showed that participants’ perceptions of nearby patients’ likely death were significantly associated with increased personal threat perceptions, *r* = 0.20, *p* < 0.001, and that increased personal threat perceptions were associated with increased 1-month PTSD score (*r* = 0.40, *p* < 0.001). Further, in the final multivariate linear regression model with personal threat perceptions entered [F(10,756) = 25.28, *p* < .001, R [2] adjusted = .24], increased personal threat perceptions during the ED visit, B = 0.71 points on the PCL per point on the personal threat perception questionnaire, β = 0.27, *p* = .001, fully mediated the association of participants’ perceptions of nearby patients’ likely death with their 1-month PTSD score (after adjustment for ED threat perceptions, B = 0.89 (95% CI, −1.33 to 3.12), β = 0.03, *p* = .43, accounting for 62% of the adjusted effect and causing the main effect of perceiving nearby patients as likely to die to become statistically nonsignificant.

## Discussion

In patients being evaluated for ACS in the ED, we tested whether perceiving that a nearby patient was likely to die was associated with greater PTSD symptom severity one month later. We found that patients who perceived that a nearby patient was likely to die had significantly greater PTSD symptoms at 1 month follow-up, and that association remained significant after adjustment for discharge diagnosis of ACS as well as demographic and clinical variables associated with PTSD risk in cardiac patients, as well as patient depression and ED crowding, which are risk factors for PTSD after ACS. We also identified a psychological mechanism for the association. Namely, when patients perceived that a nearby patient was likely to die, they were more afraid for themselves, felt less control over their own situation, more vulnerable, and more worried that they may die. These increased perceptions of personal threat fully mediated the effect, explaining 62% of the association between perceptions of nearby patients’ mortality risk and subsequent PTSD symptoms.

These findings are consistent with previous research showing that patient perception of the ED environment and the care they receive may influence psychological adjustment after evaluation for life-threatening cardiovascular events. Overcrowding, clinician-patient communication, and perceptions of a hectic ED environment have all been associated with subsequent PTSD symptoms [1, 19]. It is noteworthy that the effect of nearby patients identified in this manuscript was independent of objectively measured crowding, but ED crowding was also independently associated with 1-month PTSD symptoms.

Further, these findings support hypotheses of the Enduring Somatic Threat model of PTSD due to life-threatening medical events, which posits that fear of mortality is one of the primary risk and maintaining factors for PTSD after cardiovascular events [20]. Future work exploring the contributors to patients’ perceptions (e.g. differences in perceivers’ personality characteristics, visual or auditory cues from nearby patients or their families, activities or affective disposition of ED clinicians) may lead to the identification of modifiable factors that may improve patient ED experience and reduce secondary psychological risk.

It is important to note that, like most prior studies of PTSD in cardiac patients (see [17] for an outstanding and comprehensive review), we found no association of participant discharge diagnosis with subsequent PTSD symptoms. This further supports the notion that subjective fear and threat for individuals being treated for potentially life-threatening events in the ED may be among the most important contributors to risk for downstream psychological outcomes regardless of ultimate medical diagnosis or presumed pathophysiology leading to the ED evaluation. Although DSM 5 no longer requires peritraumatic perception of intense fear, helplessness, or horror during the index traumatic event, these results suggest that such perceptions contribute to PTSD risk in cardiac patients. Future work focusing on identifying modifiable variables that reduce subjective fear and threat for these patients may lead to decreased negative psychological outcomes such as PTSD symptoms.

Additionally, objective ED factors play an important role in shaping patient perceptions, and hospital leadership and ED clinicians can create environments that heighten or reduce stress during ED evaluation, which in turn may influence the development of PTSD symptoms after discharge. For example, efforts to minimize hallway stretcher use for patient care during periods of increased ED crowding (e.g. utilization of observation units, streamlined bed flow coordination with inpatient services) may blunt the subjective stress and subsequent development of PTSD symptom in patients evaluated under such conditions in the ED. Other initiatives such as the development of specialized geriatric EDs or sections of reduced noise volume may also be promising novel design strategies to improve psychological outcomes of patients treated for acute cardiovascular events in the ED.

There are limitations to our study that should be considered. First, the REACH study is a single-site study limited to patients presenting with ACS symptoms, so these findings may not be generalizable to all ED patients. Second, we were only able to explain 24% of the variance in PTSD scores at 1 month, which means that a great deal of variance remains unexplained. Future research should examine genetic, epigenetic, interpersonal, and physiological reactivity targets that may help to uncover these sources of variance.

A third limitation concerns measurement. Due to the practical difficulties of evaluating patients in the ED, we used a single item to assess patients’ perceptions of nearby patient mortality risk. Single item measures can be unreliable. However, by limiting the categorization of participants who perceived high acuity in neighboring patients to only those participants who reported such perception both during their ED evaluation and 3 days later, we were able to reduce the observed association of pre-existing patient factors on nearby patient perceptions. This conservative approach yielded a 12% rate of exposure to a nearby patient with high acuity, which is consistent with what would be expected given that patients were treated in a large urban academic medical center ED for a mean of 12 h. Further, the single item was correlated in the expected direction and magnitude with subsequent psychological distress and an objective crowding metric assessed concurrently. Even if the single item were unreliable, that unreliability would lead to an underestimate of the true magnitude of its association with the dependent variable.

A related issue is that we assessed participants’ perceptions of neighboring patients’ acuity and their own personal threat perceptions during the same short ED interview. Although our theoretical and statistical models imply causality, we are aware that the short time between the assessments may undercut causal claims. We argue that prior research on the influence of external evidence of threat (e.g., dead bodies during natural disasters) on subjective threat perceptions and subsequent psychological disorder supports our interpretation, and that our data collection procedures (i.e., bedside interview in the ED, so the participant must report their perception of patients that the interviewer also sees) reduce the influence of participant’s current stress on their reports of nearby patients’ status. However, the influence could be bidirectional. Future work integrating objective assessment of nearby patient acuity may provide additional information regarding the direction of influence.

With these limitations in mind, emergency physicians and staff, as well as hospital administrators and ED architects should consider our findings. For some patients in the ED, aspects of the clinical environment may be perceived as frightening and increase risk for subsequent psychological difficulties. We found that one aspect of the clinical milieu, the perception of a nearby neighboring patient being likely to die, was significantly associated with participants’ perceptions of their own vulnerability and, thereby, greater PTSD symptoms 1 month later. Future work building on our findings may guide the development of interventions for improving patient experience and secondary psychological outcomes in patients evaluated for potentially life-threatening cardiovascular events in the ED.

## Conclusion

Patients’ experiences in the emergency department during evaluation for an acute life-threatening cardiac event may influence their subsequent psychological health after hospital discharge. Our research found that patients’ perceptions of this setting, specifically proximity to another patient perceived as mortally ill, can influence the development of PTSD symptoms 1 month after the event. This research highlights the importance of ED environment factors for patients’ perceptions of their own personal threat and subsequent psychological adjustment. Future research should examine additional factors that influence this perception of threat and determine whether modifying ED environments reduce psychological risk.

## Abbreviations

ACS: Acute coronary syndrome
ED: Emergency department
EDWIN: Emergency department work index
EST: Enduring somatic threat model
MI: Myocardial infarction
NSTEMI: Non-ST elevation myocardial infarction
PHQ: Patient health questionnaire
PTSD: Posttraumatic stress disorder
UA: Unstable angina
